# Enhancing Hi-C data resolution with deep convolutional neural network HiCPlus

**DOI:** 10.1038/s41467-018-03113-2

**Published:** 2018-02-21

**Authors:** Yan Zhang, Lin An, Jie Xu, Bo Zhang, W. Jim Zheng, Ming Hu, Jijun Tang, Feng Yue

**Affiliations:** 1 Department of Computer Science and Engineering, University of South Carolina, Columbia, SC 29208 USA; 2 Bioinformatics and Genomics Program, Huck Institutes of the Life Sciences, The Pennsylvania State University, University Park, PA 16802 USA; 3 Department of Biochemistry and Molecular Biology, College of Medicine, The Pennsylvania State University, Hershey, PA 17033 USA; 4 School of Biomedical Informatics, University of Texas Health Science Center at Houston, Houston, TX 77030 USA; 5 Department of Quantitative Health Sciences, Lerner Research Institute, Cleveland Clinic Foundation, Cleveland, OH 44195 USA; 6 School of Computer Science and Technology, Tianjin University, 300072 Tianjin, China; 7 Tianjin University Institute of Computational Biology, Tianjin University, 300072 Tianjin, China

## Abstract

Although Hi-C technology is one of the most popular tools for studying 3D genome organization, due to sequencing cost, the resolution of most Hi-C datasets are coarse and cannot be used to link distal regulatory elements to their target genes. Here we develop HiCPlus, a computational approach based on deep convolutional neural network, to infer high-resolution Hi-C interaction matrices from low-resolution Hi-C data. We demonstrate that HiCPlus can impute interaction matrices highly similar to the original ones, while only using 1/16 of the original sequencing reads. We show that the models learned from one cell type can be applied to make predictions in other cell or tissue types. Our work not only provides a computational framework to enhance Hi-C data resolution but also reveals features underlying the formation of 3D chromatin interactions.

## Abstract (web summary)

Despite its popularity for measuring the spatial organization of mammalian genomes, the resolution of most Hi-C datasets is coarse due to sequencing cost. Here, Zhang et al. develop HiCPlus, a computational approach based on deep convolutional neural network, to infer high-resolution Hi-C interaction matrices from low-resolution Hi-C data.

## Introduction

The high-throughput chromosome conformation capture (Hi-C) technique^1^ has emerged as a powerful tool for studying the spatial organization of chromosomes, as it measures all pair-wise interaction frequencies across the entire genome. In the past several years, Hi-C technique has facilitated several exciting discoveries, such as A/B compartment^1^, topological associating domains (TADs)^2,3^, chromatin loops^4^, and frequently interacting regions (FIREs)^5^, and therefore significantly expanded our understanding of three-dimensional (3D) genome organization^1,2,4^ and gene regulation machinery^6^. Hi-C data are usually presented as an *n* × *n* contact matrix, where the genome is divided into *n* equally sized bins and the value within each cell of the matrix indicates the number of pair-ended reads spanning between a pair of bins. Depending on sequencing depths, the commonly used sizes of these bins can range from 1 kb to 1 Mb. The bin size of Hi-C interaction matrix is also referred to as 'resolution', which is one of the most important parameters for Hi-C data analysis, as it directly affects the results of downstream analysis, such as predicting enhancer–promoter interactions or identifying TAD boundaries. Sequencing depth is the most crucial factor in determining the resolution of Hi-C data—the higher the depth, the higher the resolution (smaller bin size).

Owing to high sequencing cost, most available Hi-C datasets have relatively low resolution such as 25 or 40 kb, as the linear increase of resolution requires a quadratic increase in the total number of sequencing reads^6^. These low-resolution Hi-C datasets can be used to define large-scale genomic patterns such as A/B compartment or TADs but cannot be used to identify more refined structures such as sub-domains or enhancer–promoter interactions. Therefore, it is urgent to develop a computational approach to take full advantage of these currently available Hi-C datasets to generate higher-resolution Hi-C interaction matrix.

Recently, deep learning has achieved great success in several disciplines^7–9^, including computational epigenomics^10–13^. In particular, Deep Convolutional Neural Network (ConvNet)^7,14^, which is inspired by the organization of the animal visual cortex^14–16^, has made major advancement in computer vision and natural language processing^7^. In the fields of computational biology and genomics, ConvNet has  been successfully implemented to predict the potential functional of DNA sequence^17–22^, DNA methylation or gene expression patterns^23–26^.

In this work, we propose HiCPlus, which is the first approach to infer high-resolution Hi-C interaction matrices from low-resolution or insufficiently sequenced Hi-C samples. Our approach is inspired by the most recent advancements^27–30^ in the single image super-resolution and can generate the Hi-C interaction matrices with the similar quality as the original ones, while using as few as 1/16 of sequencing reads. We observe that Hi-C matrices are composed by a series of low-level local patterns, which are shared across all cell types. We systematically applied HiCPlus to generate high-resolution matrices for 20 tissue/cell lines (Supplementary Table 1) where only low-resolution Hi-C datasets are available, covering a large variety of human tissues. In summary, this work provides a great resource for the study of chromatin interactions, establishes a framework to predict high-resolution Hi-C matrix with a fraction of sequencing cost, and identifies potential features underlying the formation of 3D chromatin interactions.

## Results

### Overview of HiCPlus framework

**Fig. 1:**
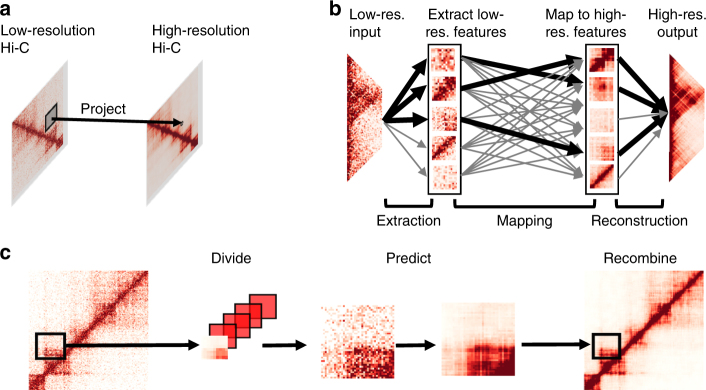
Overview of the HiCPlus pipeline. **a** HiCPlus leverages information from surrounding regions to estimate contact frequency for a given point in a Hi-C interaction matrix. **b** Conceptual view of the network structure in HiCPlus: regional interaction features (e.g., loops, domain borders) are learned using values at each position in the high-resolution matrix as the response variable and using its neighbouring points from the low-resolution matrix as the predictors. **c** HiCPlus divides the entire Hi-C matrix into small square samples and enhances them separately. After each block of interactions are predicted, those blocks are merged into chromosome-wide interaction matrix

Figure 1 illustrates the overall framework of HiCPlus. To train the ConvNet model, we first generate a high-resolution matrix (10 kb) with deeply sequenced Hi-C data, such as those from GM12878 or IMR90 cells. Next, we down-sample the sequencing reads to 1/16 and construct another interaction matrix at the same resolution, which consequently contains more noises and more blurred patterns. We then fit the ConvNet model using values at each position in the high-resolution matrix as the response variable and using its neighbouring points from the down-sampled matrix as the predictors (Fig. 1a). Our goal is to investigate whether the ConvNet framework can accurately predict values in the high-resolution matrix using values from the low-resolution matrix. Noticeably, although technically both matrices are at the same resolution, we consider the down-sampled interaction matrix 'low resolution', as in practice, it is usually processed at lower resolution due to the shallower sequencing depths. In this paper, we use 'low-resolution' and 'insufficiently sequenced' interchangeably.

We describe the conceptual view of the ConvNet in Fig. 1b, which learns the mapping relationship between high-resolution Hi-C matrix and low-resolution Hi-C matrix at feature levels. Once the model is trained, we can apply it to enhance any Hi-C interaction matrix with low-sequencing depth. HiCPlus divides the entire Hi-C matrix into small square samples and enhance them separately. After each block of interactions are predicted, those blocks are merged into chromosome-wide interaction matrix Fig. 1c. The detailed structure of ConvNet is described in Supplementary Fig. 1 and more detailed description of the algorithm is described in the Methods section.

### Chromatin interactions are predictable from neighbouring regions

Our hypothesis is that the Hi-C matrix contains repeating local patterns, and the interaction intensity of each point is not independent to its local neighbouring regions. Therefore, we should be able to predict the interaction frequency of any cell in the Hi-C matrix with the interaction frequencies from its neighbouring regions. To test this hypothesis, we trained a ConvNet model on chromosomes 1–17 and systematically predicted interaction matrices in chromosomes 18–22, using the 10 kb resolution Hi-C data in GM12878 cells^4^. To evaluate the performance of our ConvNet model, we computed both the Pearson and Spearman correlation coefficients between the predicted values and the real values at each genomic distance.

**Fig. 2:**
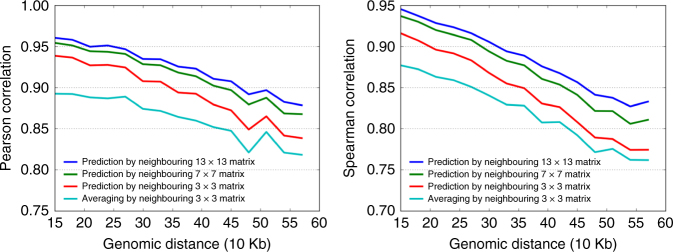
Predicting chromatin interactions from their neighbouring regions. We trained a ConvNet model on chromosomes 1–17 and systematically predicted interaction matrices in chromosomes 18–22, using the 10 kb resolution Hi-C data in GM12878 cell line. We used three surrounding regions sizes (3 × 3, 7 × 7, 13 × 13) for prediction and also compared their performances with a naive prediction method that simply averages the neighbouring 3 × 3 matrix. We observe that using 13 × 13 matrix achieve the best performance at each genomic distance when evaluated by both Pearson and Spearman correlations

An important parameter in our model is the size of neighbouring regions: intuitively, to predict the value of one point, using a larger surrounding matrix will yield better results. Therefore, we tested a range of neighbouring matrix sizes, from 3 × 3 to 15 × 15. Indeed, we observed that using a larger neighbouring matrix generally increases the prediction accuracy. When using a 13 × 13 surrounding matrix, the Pearson correlations between the predicted and real interaction frequencies are consistently higher than the predictions using smaller surrounding matrices, at each genomic distance. For example, the Pearson correlation at 40 kb genomic distance for 13 × 13, 7 × 7 and 3 × 3 matrices are 0.93, 0.92, and 0.89, respectively (Fig. 2). However, we found that the prediction accuracy reached a plateau when we used the 13 × 13 matrix prediction model, and further increasing the size of surrounding matrix shows little if any improvement of the prediction accuracy (Supplementary Fig. 2).

For comparisons, we first tried a simple approach, by predicting each interaction frequency using the average values from its surrounding matrix. After testing a series of block sizes, we found that 3 × 3 gives the best performance for averaging-based method (Supplementary Fig. 3). Next, we compared the ConvNet with a few other commonly used methods, such as two-dimensional (2D) Gaussian smoothing and Random Forest. We observed that ConvNet performs the best among all these methods (Fig. 2).

Finally, we compared the performance of training one model for the whole matrix with training a model for each genomic distance. It is known that there is distance decay in the Hi-C interaction matrix, which means that the further away a bin is from the diagonal, the smaller value it tends to be. Therefore, we trained a set of models and each model is used for prediciting chromatin interactions at one given distance (e.g. 10 kb, 20 kb, …). However, this approach did not improve the prediction accuracy (Supplementary Fig. 2), indicating that our current model has incorporated the distance effect and it is not necessary to train different models at different genomic distances.

### Enhancing chromatin interaction matrix with low-sequence depth

**Fig. 3:**
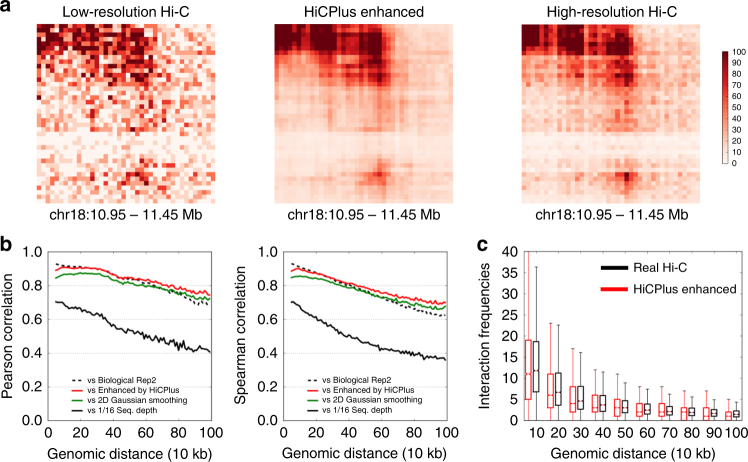
HiCPlus accurately enhances interaction matrix with low-sequence depth. We trained the model on chromosomes 1–8 and tested the prediction in chromosome 18 in the same cell type (GM12878) at 10 kb resolution. For prediction, we random chose 1/16 reads from the original total reads, built an interaction matrix (**a**, left panel) and then used HiCPlus to enhance it (**a**, middle panel). **a** HiCPlus-enhanced Hi-C and real high-resolution Hi-C matrices are highly similar. **b** High correlations between HiCPlus-enhanced and real high-resolution Hi-C matrices at each genomic distance, and they are close to the correlations between two biological replicates (dotted line). We also observed that 2D Gaussian smoothing also performs well but not as well as HiCPlus. Their correlations with the down-sampled Hi-C matrix is much lower (solid blue line). **c** Distribution of the Hi-C interaction frequencies at each distance for real Hi-C and HiCPlus-enhanced matrices are similar. The whiskers are 5 and 95 percentile

Having established that values in Hi-C matrix can be predicted using their surrounding regions, we then investigated whether we can predict these values with insufficiently sequenced samples. We first trained and tested our HiCPlus model in the same cell type, using the high-resolution Hi-C data in GM12878 cell (access code GSE63525)^4^. We constructed the 10 kb resolution matrix using all the reads (Fig. 3a, right panel). Then we down-sampled the reads to 1/16 of the original sequencing depth and constructed the interaction matrix at the same resolution (Fig. 3a, left panel). The newly generated matrix contains lots of noise and TAD structures are less clear. Next, we fit a ConvNet model using values at each bin on the high-quality matrix as the response variable and using its neighbouring 13 × 13 points in the down-sampled matrix as predictors. We used chromosomes 1–7 as the training datasets and chromosome 13 as the validation set to obtain the optimal hyperparameters. Then we applied it to enhance the down-sampled interaction matrix in chromosome 18. An example of a ConvNet-enhanced matrix is shown in Fig. 3a (middle panel). We observed that the HiCPlus-enhanced matrix is highly similar with the real high-resolution Hi-C matrix. Compared with the matrix generated from down-sampled reads, it contains much less noise and both the individual chromatin loops and the TAD structures are more visible.

To quantitatively evaluate the performance of HiCPlus, we computed the Pearson correlation and Spearman ranking correlation between the experimental high-resolution matrix, down-sampled matrix, 2D Gaussian Smoothing-enhanced and HiCPlus-enhanced matrix at each genomic distance. As shown in Fig. 3b and Supplementary Fig. 4, the HiCPlus-enhanced matrix obtained much higher correlation with the real high-resolution Hi-C matrix than the down-sampled matrix at all genomic distances. Surprisingly, the correlations between the HiCPlus-enhanced matrix and the real high-resolution Hi-C matrix are nearly as high as those between two real high-resolution matrices from two biological replicates in the same cell type (Fig. 3b), suggesting that ConvNet framework can reconstruct a high-resolution interaction matrix using only a fraction of the total sequencing reads. We thoroughly tested the model and found that its performance of the model is consistent across different chromosomes (Supplementary Fig. 5).

To compare deep convolutional neural network with other approaches, we first implemented several image denoising methods, including 2D Gaussian smoothing, 2D average smoothing and anisotropic diffusion (Supplementary Fig. 6). We tested and selected the best parameters for 2D Gaussian smoothing (Supplementary Fig. 7) and used suggested parameters for 2D Average smoothing from previous work^31^. To compare with non-deep learning frameworks, we also implemented a Random Forest Regressor, using the default parameters from Sklearn^32^. Among all the methods, we observe that HiCPlus has the best performance, followed by Gaussian smoothing and Radom Forest (Fig. 3, Supplementary Fig. 6).

It has been shown that there are systematic biases in Hi-C data^33,34^, such as GC contents, number of cutter sizes and mappability. Applying systematic normalization can remove these biases and generate more accurate contact maps. Therefore, we also investigated whether HiCPlus can be used to enhance the normalized Hi-C matrix. As shown in Supplementary Fig. 8, HiCPlus can be also applied to increase the resolution of normalized Hi-C matrix.

### Enhancing Hi-C interaction matrices across different cell types

**Fig. 4:**
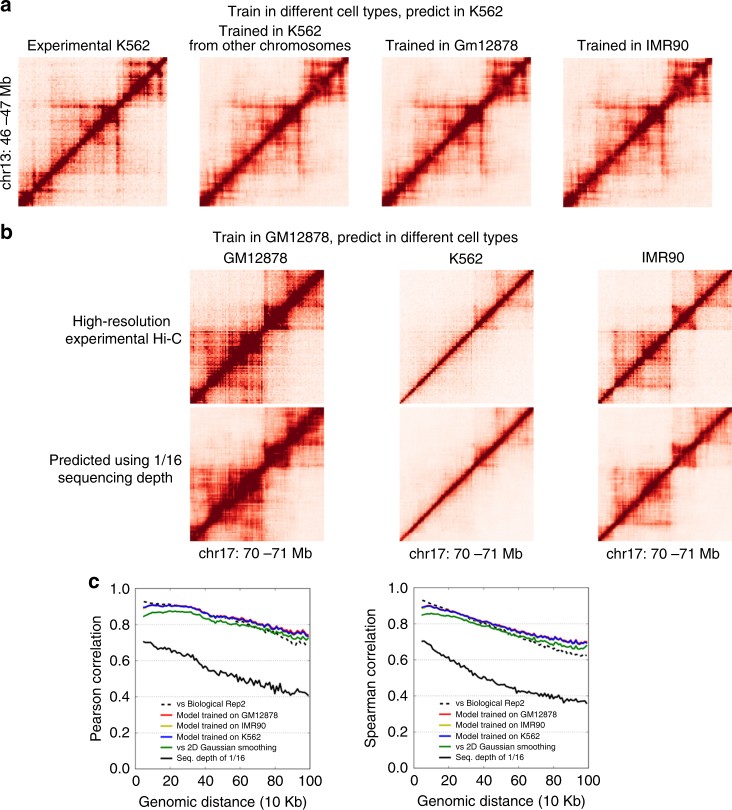
HiCPlus can learn model from one cell type and predict in other cell types. Shown in the figures are real and HiCPlus-enhanced matrices in GM12878, K562 and IMR90 at 10 kb resolution. **a** HiCPlus-enhanced matrices in K562 with models trained in three different cell types are highly similar to each other, and all of them are also similar to the original K562 interaction matrix. **b** Model trained in GM12878 can be used to predict interaction matrices in different cell types (K562, GM12878 and IMR90). **c** High correlations between HiCPlus-enhanced matrices using models trained in three different cell types, 2D Gaussian kernel and high-resolution Hi-C at each genomic distance

A key application for HiCPlus is to enhance the resolution of existing low-resolution Hi-C matrices from the previous studies^2,35–44^ with the model trained on the cell types where high-resolution Hi-C data are available^4,38^. The results can also be used to address whether the low-level local patterns on Hi-C matrix are shared across different cell types as well. First, we trained the ConvNet model in three different cell types (GM12878, K562, IMR90)^4^ with similar sequencing depths and tested their prediction performances in K562 cells. Similar to the procedure showed in the previous section, we down-sampled Hi-C reads in K562 to 1/16 and then applied ConvNet to enhance its interaction matrix. As shown in Fig. 4a, the enhanced Hi-C matrices using three different training datasets are highly similar to each other. More importantly, all of them are also similar to the original high-resolution interaction matrix (Figs. 4a, c), suggesting that the local patterns/features captured by ConvNet framework from different Hi-C matrices are highly similar and can be used to enhance Hi-C matrix in other cell types.

To further validate this observation, we trained the ConvNet model in GM12878 cells and applied it to enhance Hi-C matrices in three different cell types (GM12878, K562, IMR90). Again, we found that the ConvNet-enhanced Hi-C matrices are highly similar to the real high-resolution Hi-C matrices. An example is shown in Fig. 4b, where the chromatin interaction patterns in this region are highly dynamic across different cell types. However, the ConvNet-enhanced matrices accurately depict these differences and help demonstrating these cell-type-specific TADs and chromatin interactions. Finally, we applied HiCPlus and systematically enhanced the low-resolution Hi-C interaction matrices previously generated in 20 tissues/cell types^2,35–44^.

To predict such datasets, in the first step, we trained models for different sequencing depth from (×4 to ×16). Then we generate the 10 kb Hi-C interaction matrix from the BAM file in Hi-C library. In order to determine the enhancement scale, we calculate the ratio of the effective sequencing depth between the candidate Hi-C matrix and Hi-C training matrix between genomic distances of 25,000 to 1,000,000 base pairs. If the sequencing depth of candidates’ Hi-C matrix is <1/16 of training Hi-C matrix, we use the ×16 model.

### Identifying chromatin interactions in HiCPlus-enhanced matrices

**Fig. 5:**
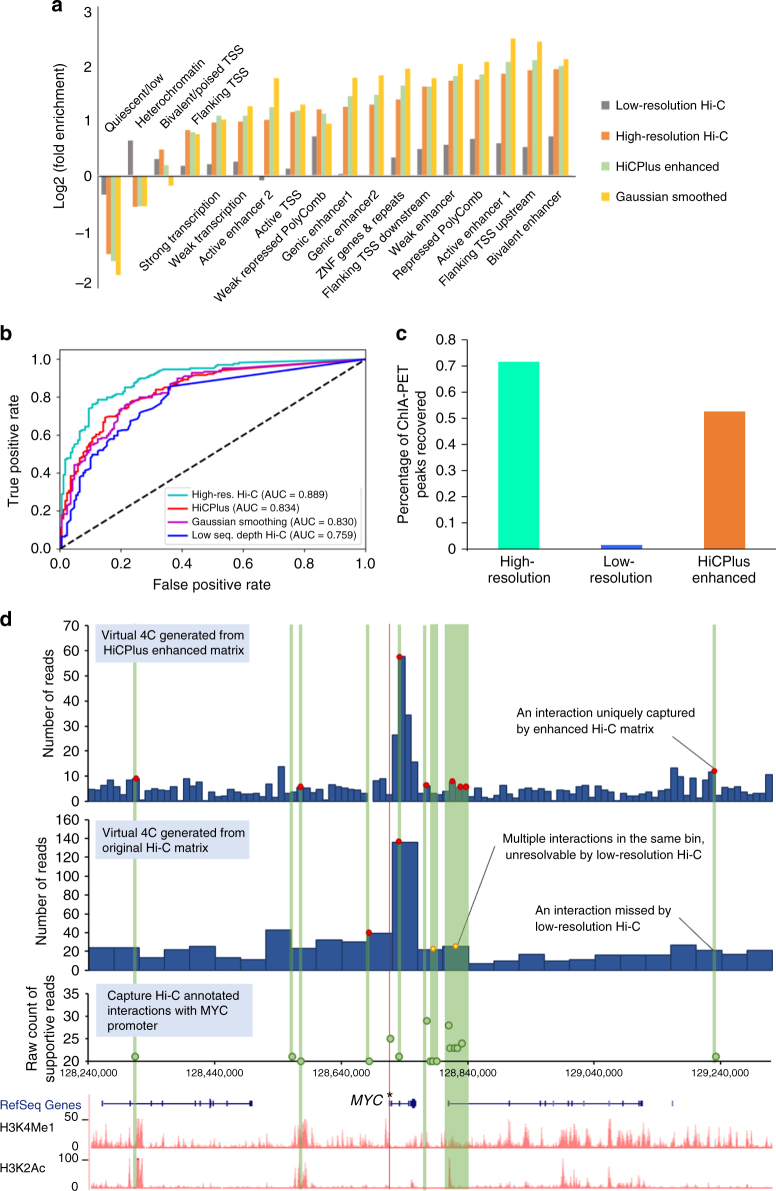
Analysis of chromatin interactions identified in the HiCPlus-enhanced matrix. **a** We observe that the chromatin loops in HiCPlus-enhanced and real high-resolution interaction matrices are enriched for the same categories of functional elements and the enrichment level are similar. While Gaussian kernel-enhanced matrix is enriched for the same categories of elements, the levels are different from those in high-resolution Hi-C matrix. Chromatin loops were predicted by Fit-Hi-C with a stringent cutoff (*q*-value < 1e-06) in down-sampled Hi-C, HiCPlus-enhanced, Gaussian kernel-enhanced and real high-resolution Hi-C matrices in K562 cell line at 10 kb resolution. The functional annotations by chromHMM are downloaded from the Roadmap project. Enrichment levels are computed as fold change (log2 converted) against their distribution across the whole genome. **b** ROC analysis of overlaps between interactions from CTCF ChIA-PET with identified interacting peaks from down-sampled Hi-C, HiCPlus-enhanced, Gaussian kernel-enhanced and real high-resolution Hi-C matrices in K562 cell line. **c** Percentage of CTCF ChIA-PET peaks that overlap with chromatin interactions identified in real high-resolution, down-sampled and HiCPlus-enhanced matrices. **d** HiCPlus-enhanced matrix captures interactions between *MYC* promoter and *cis*-regulatory elements that are missed or unresolved by low-resolution Hi-C matrix. The top two virtual 4C tracks are generated using HiCPlus-enhanced matrix (10 kb resolution) and the original matrix (40Kb resolution) from aorta tissue, anchored on *MYC* promoter (marked by asterisk). We compared virtual 4C tracks with Capture Hi-C data in the same region, supported by at least 20 reads in GM12878 cells. Red dots indicate the Capture Hi-C peaks that are also detected by Hi-C. We notice that multiple Capture Hi-C interactions are mapped to the same 40 kb bin and thus unresolvable by the low-resolution Hi-C matrix (yellow dots in the low-resolution virtual 4C). However, these interactions are captured by the HiCPlus-enhanced matrix. We also notice that these interactions are between *MYC* promoter and potential distal enhancers, marked by H3K4me1 and H3K27ac

It has been shown that strong chromatin interactions (loops) are enriched for important regulatory elements, such as enhancers and promoters^4^. After demonstrating that HiCPlus can transform low-resolution Hi-C data to high-resolution Hi-C interaction matrix, we investigated whether these enhanced high-resolution matrices can facilitate the identification of meaningful chromatin interactions. For this purpose, we used the Fit-Hi-C^45^ software, which can adjust random polymer looping effect and estimate statistical confidence of intra-chromosomal interactions. We applied Fit-Hi-C to the real high-resolution, 1/16 down-sampled and HiCPlus-enhanced interaction matrices at 10 kb resolution in K562 cell line, respectively. We kept the predicted significant interactions (*q*-value < 1e-06) in genomic distance from 30 to 500 Kb for further comparative analysis. We first observed that 72.37% (6340/8760) of the peaks identified in the HiCPlus-enhanced matrix were also identified in the true high-resolution matrix (Supplementary Fig. 9, Supplementary Table 2). Then we investigated whether the predicted chromatin interactions from three matrices are enriched for potential functional elements annotated by ChromHMM^46^. As shown in Fig. 5a, significant interactions from the real high-resolution Hi-C matrix and HiCPlus-enhanced matrix show similar patterns: enriched for active states, such as enhancer-associated states (‘Weak Enhancer’, ‘Active Enhancer 1&2’, ‘Bivalent Enhancer’ and ‘Genic enhancer1&2’) and promoter-associated states (‘Flanking TSS Upstream’, ‘Flanking TSS Downstream’ and ‘Active TSS’), while depleted of inactive states, such as quiescent and heterochromatin-associated states (‘Quiescent/Low’ and ‘Heterochromatin’). On the contrary, the interactions identified in the down-sampled Hi-C matrix show discrepant patterns with those identified in real high-resolution Hi-C matrix. For example, they are enriched for heterochromatin and minimal if any enrichment of active transcription start site (TSS), suggesting that interactions identified from the down-sampled matrix are of poor quality and might give false information if analysed at this resolution^47^.

Next, we compared the predicted chromatin interactions from the real high-resolution Hi-C, down-sampled Hi-C and HiCPlus-enhanced matrices with the identified chromatin loops by CTCF chromatin interaction analysis by paired-end tagging sequencing (ChIA-PET) in the same cell type. We used the identified CTCF-mediated chromatin loops from ChIA-PET as true positives. As for negatives, we randomly selected the same number of pairs of CTCF-binding sites that are not predicted as interacting pairs by ChIA-PET (Methods). Then we plotted the AUC (area under the curve) to evaluate the performance of our model. As shown in Fig. 5b, CTCF interacting pairs and non-interacting pairs are separated in the predicted results from HiCPlus-enhanced matrix (average AUC = 0.85). We also observed that the AUC score for the HiCPlus-enhanced matrix is significantly higher than the AUC from the down-sampled matrix (*p*-value < 0.05). Finally, we compared the overlap between significant interactions identified in three interaction matrices with the ChIA-PET identified interactions (Fig. 5c). Seventy-nine and 67% of ChIA-PET-predicted loops can be recovered by interactions identified in the real and HiCPlus-enhanced matrices, respectively, while only 9% of the ChIA-PET interactions can be recovered by the down-sampled Hi-C matrix, demonstrating again that by HiCPlus-enhanced matrix the analysis of the down-sampled matrix at this resolution is not reliable. To further show the power of HiCPlus framework, we applied it to enhance the Hi-C dataset from aorta tissue where only low-resolution (40 kb) matrices are available (Fig. 5d). By comparing chromatin interactions from Capture Hi-C, we observe that HiCPlus-enhanced matrix captures significant interactions between *MYC* promoter and *cis*-regulatory elements that are missed or unresolved by low-resolution Hi-C matrix. For example, multiple Capture Hi-C interactions are mapped to the same 40 kb bin and thus unresolvable by the low-resolution Hi-C matrix (yellow dots on the second 4C track). However, these interactions are captured by the enhanced matrix, suggesting that HiCPlus can improve the resolution of Hi-C interaction matrix and reveal meaningful interactions that are missed by original low-resolution Hi-C data.

In summary, the ConvNet framework can significantly improve the quality of interaction matrix for insufficiently sequenced Hi-C samples and further facilitate identifying biologically meaningful interactions that are enriched for potential functional elements and validated by other techniques.

## Discussion

Here we present HiCPlus, the first computational approach to infer high-resolution Hi-C interaction matrices from low-resolution Hi-C data. Our framework can construct the interaction matrix with similar quality using only 1/16 or even fewer sequencing reads. We systematically applied HiCPlus to generate high-resolution matrices for 20 tissue/cell types where only low-resolution Hi-C data are available, covering a large variety of human tissues.

We observe that Hi-C interaction matrices are composed of a series of low-level repeating local patterns, which are shared across all cell types and tissues. These features can be effectively captured by our ConvNet framework and used to enhance Hi-C matrix in different cell types. However, most of these local patterns are still represented as black boxes in the intermediate convolutional layers and therefore are not human interpretable. We hypothesize that these features are related to important functions in 3D genome organization, such as chromatin loops and TADs. More work on visualizing and interpreting these features are imperative and will be of great values to deepen our understanding of the high-order genome organization and gene regulation.

Another caveat is the ground truth used for training and evaluating in the ConvNet framework. Throughout the analyses in this work, we used the real high-resolution Hi-C matrix as the ground truth/gold standard. However, there are natural variations even between high-resolution interaction matrices from different biological replicates in the same cell type. In the functional enrichment analysis (Fig. 5a), the significant interactions in the ConvNet-enhanced matrix are more enriched for some epigenetic markers than those from the real high-resolution Hi-C matrix. In addition, previous work from other disciplines^8,47,48,49^ have reported that introducing noises in the training process can increase the prediction accuracy of the deep learning model. It is possible that the deep ConvNet model can distinguish noises from real signals in the Hi-C matrices, which contributes to the interaction matrix enhancement. Indeed, we observe that HiCPlus performs better in identifying the significant chromatin interactions than other methods (Supplementary Fig. 10). Further investigations are needed to validate and interpret these interesting observations and the results might shed light on how to improve the computational model and deepen our understanding of chromatin interactions.

It is interesting that the performance of Gaussian kernel and deep learning have comparable results, especially when evaluated by genome-wide correlation. In this project, our primary goal is to enhance low-resolution Hi-C data matrix to higher-resolution Hi-C data, which is essentially an image-enhancing problem. Therefore, it is not surprising that Gaussian kernel and diffusion-based methods have solid performance, as they have been widely used in solving such problems. On the other hand, as shown in Supplementary Fig. 10, we also observe that deep-learning framework performs better at significant chromatin interaction regions and that HiCPlus predicted values are closer to the values in experimental high-resolution Hi-C matrix. Finally, although Gaussian smoothing can provide solid performance for enhancing Hi-C matrix, it will not provide any additional biologically meaningful information. HiCPlus, on the other hand, is trained by learning certain patterns and information from the training datasets, and these patterns are used for enhancing Hi-C matrix in the prediction process. In future studies, we will further study these patterns and hopefully we can recover more biologically meaningful interpretation of the results.

In summary, HiCPlus presents the first deep learning framework for enhancing the resolution of Hi-C interaction matrices. By leveraging interaction frequencies from neighbouring regions and learning regional patterns from available high-resolution Hi-C data, HiCPlus can generate high-resolution Hi-C interaction matrices at a fraction of the original sequencing reads. With the fast accumulation of Hi-C data in different cell lines and tissue types, we provide a rich resource and a powerful tool for the study of 3D genome organization and gene regulation.

## Methods

### HiCPlus workflow

Step 1, Pre-processing Hi-C matrix: Many of the current available Hi-C data, especially in human tissues^2,5,35^, are only available at 40 kb resolution matrices. For these data sets, we start from the BAM file and generate the 10 Kb resolution interaction matrices. Consequently, we observe an increased noise-to-signal ratio comparing with deeply sequenced Hi-C library. In the training stage, we start from high-resolution Hi-C data (such as GM12878 from GSE63525) and use a random down-sampling method to simulate the low-resolution Hi-C matrix. After this step, all input matrices are at 10 Kb resolution. As previously mentioned, we consider the matrices generated from down-sampled sequencing reads as low resolution since they would have been processed at a lower resolution at that sequencing depths in practice.

Step 2: Divide a Hi-C matrix into multiple square-like sub-regions with fixed size, and each sub-region is treated as one sample. Unless otherwise noticed, each sub-region is 0.4 × 0.4 Mb^2^, which contains 40 × 40 = 1600 pixels at 10 Kb resolution. We only investigate and predict chromatin interactions where the genomic distance between two loci is <2 Mb, as the average size of TADs is <1 Mb and there are few meaningful interactions outside TADs.

Step 3: The deep ConvNet is trained to learn the relationship between the low-resolution samples (a.k.a., same size but insufficient sequenced samples) and high-resolution samples in the training stage and predicts the high-resolution samples from low-resolution samples in the production stage.

Step 4: The predicted high-resolution sub-matrices are merged into chromosome size Hi-C interaction matrix. As the samples have a surrounding padding region that is removed during the prediction by ConvNet, the proper overlap is necessary when dividing the Hi-C interaction matrix to the samples in the Step 1.

### ConvNet structure

For the ConvNet, the input is a list of low-resolution samples with *N* × *N* size for each sample. To avoid the border effect, similar with Dong’s work^30^, we did not add white padding to any convolutional layer so the output of each sample has the smaller size. Therefore, the output is a list of predicted high-resolution samples with (*N*−padding) × (*N*−padding) size, where *N* = 40 and padding = 12 for the typical setting in this discussion, and both input 40 × 40 matrix and output 28 × 28 matrix are registered in the same central location. The shrunk size can be offset by the overlapping during the dividing process, *X*_*i*_.

We denote the ConvNet model as *F*, the low-resolution input as *X*, the predicted high-resolution output as *Y* and the real high-resolution Hi-C as *Y* (*Y* is also regarded as ground truth in this section). Mean square error (MSE) is used as loss function in the training process. Therefore, the goal of the training process is to generate *F* that minimizes the MSE.

$$\mathrm{argmin}\frac{1}{\it m}\mathop {\sum}\nolimits_{i = 1}^{\it m} {\left\| {{\it F}\left( {{\it X}{\it i}} \right) - {\it Y}} \right\|^2}$$

As shown in Fig. 1b, the ConvNet in HiCPlus has three layers, serving for extracting and representing patterns on the low-resolution matrix, non-linearly mapping the patterns on the low-resolution matrix to high-resolution matrix and combining the high-resolution patterns to generate the predicted matrix, respectively. Below we describe each layer in detail.

### Pattern extraction and representation

In this stage, input is the low-resolution *f*_1_ × *f*_1_ matrix, and output is generated by the following formula

$$F1\left( X \right) = {\mathrm{max}}\left( {0,\,w_1 \ast X + b_1} \right)$$

where * denotes the convolutional operation, *X* is the input matrix, *b*_1_ is the bias and *w*_1_ is an *n*_1_ × *f*_1_ × *f*_1_ matrix. Here *n*_1_ and *f*_1_ are the filter numbers and filter size, respectively. Both *n*_1_ and *f*_1_ are hyperparameters in the ConvNet, and we set *n*_1_ to 16 and *f*_1_ to 5. As shown in (Supplementary Fig. 1c), HiCPlus is not sensitive to these hyperparameters. The Rectified Linear Unit (ReLU)^50^ is utilized as the non-linear activation function.

### Non-linear mapping between the patterns on high-and low-resolution maps

This stage is shown as the middle part of the Fig. 1b, where the patterns on the low-resolution matrix are mapped non-linearly with the patterns on high-resolution matrix using the formula:

$$F2(X) = {\mathrm{max}}\left( {0,\,w_2 \ast F1(X) + b_2} \right)$$

where *F*1(*X*) is the output from the previous layer, *b*_2_ is the bias and *w*_2_ are *n*_2_ matrices, each has the size of *f*_2_ × *f*_2_. In this layer, we set *n*_2_ to 16 and *f*_2_ to 1 as it is a process of non-linear mapping.

### Combining patterns to predict high-resolution maps

We employ the following formula to generate the predicted high-resolution Hi-C matrix from the results of the second layer

$$F3(X) = w_3 \ast F2(X) + b_3$$

where *F*2(*X*) is the output from the previous layer, *b*_3_ is the bias and *w*_3_ are *n*_3_ matrices of size *f*_3_ × *f*_3_. In this step, the non-linear activation function is not required, and the filter number *n*_3_ is set to 1 to generate the predicted results.

Overall, function *F* has parameters *Θ* = {*w*_1_, *w*_2_, *w*_3_, *b*_1_, *b*_2_, *b*_3_}. The goal of the training process is to obtain the optimal *Θ* to minimize MSE on the samples in the training set. We employ the standard backpropagation^14^ with gradient descent to train the network and use Stochastic Gradient Descent^51^ as the update strategy. The initial parameters are drawn from the uniform distribution with Glorot’s strategy^52^ unless otherwise noted. The training is converged and no over-fitting is observed (Supplementary Fig. 11).

We noted that sequencing depth has great impact on the performance of HiCPlus. In this work, to make enhanced matrices for the 20 human tissue/cell types, we trained three models in IMR90 cell lines, depending on the sequencing depth of the sequenced Hi-C data in different tissue/cell type: >80 million, 50–80 million, <50 millions (more detailed breakdown in Supplementary Table 1). All three models were trained on chromosomes 1–8 and tested in chromosome 18. To achieve the best result, an individual user is recommended to retrain the model according to the sequencing depth. The user can simply down-sample the Hi-C reads in GM12878 or IMR90 to match their read numbers and run our pipeline to train their model.

### Testing hyperparameter settings for HiCPlus

To find the optimal setting for HiCPlus, we have implemented the convolutional neural network with two layers and another model with three layers without ReLU activation. We observed that the performances of these three models (two layers vs. three layers without ReLU vs. three layers with ReLU) are almost indistinguishable, when evaluated by correlations at each distance between enhanced and original matrices (Supplementary Fig. 12, red, blue and yellow dashed line). However, when we further zoomed in and inspected individual chromatin loops, we noticed that, compared to the network with the three-layer and ReLU activation, the other two models show reduced chromatin intensities at chromatin loop regions (white circles in Supplementary Fig. 13a and 13b). For example, the colours of the high-resolution and HiCPlus matrices are both red (high intensity), while the colours in the other two models are 'blue' (low intensity). We show another similar example in Supplementary Fig. 13b.

We also evaluated whether pooling layers could help improve the performance of HiCPlus. We tried both maximum pooling and mean pooling and showed the results in Supplementary Fig. 14. We did not notice any difference in performance: the correlation from the models with pool layers is no better than the model without pooling. Therefore, there is no obvious advantage using a pooling layer.

In the current implementation of the software, we did not use the interpolation step and directly processed the low-resolution Hi-C data and generated the interaction matrix, using the same number of bins as the high-resolution Hi-C. We also tried to use the low-resolution interpolated matrix but observed that the interpolation did not perform as well as directly using the low-resolution matrix. As shown below (Supplementary Fig. 15,16), simple bicubic interpolation (yellow line) and the bicubic interpolation followed by ConvNet (black line) both have good performance but not as good as HiCPlus.

### Data availability

Source code is publicly available at available in the GitHub repository (https://github.com/zhangyan32/HiCPlus). Enhanced Hi-C datasets can be downloaded from http://promoter.bx.psu.edu/public/HiCPlus/matrix/ and can be visualized in the 3D Genome Browser (http://3dgenome.org).

## Electronic supplementary material

Supplementary Information
