# Supplementary material for: Enhancing Hi-C data resolution with deep convolutional neural network HiCPlus
Source: Nat Commun. 2018 Feb 21;9:750. doi: 10.1038/s41467-018-03113-2 (PMC5821732; doi:10.1038/s41467-018-03113-2)
Supplement: Supplementary file 1 — Supplementary Information [file 41467_2018_3113_MOESM1_ESM.pdf]

a

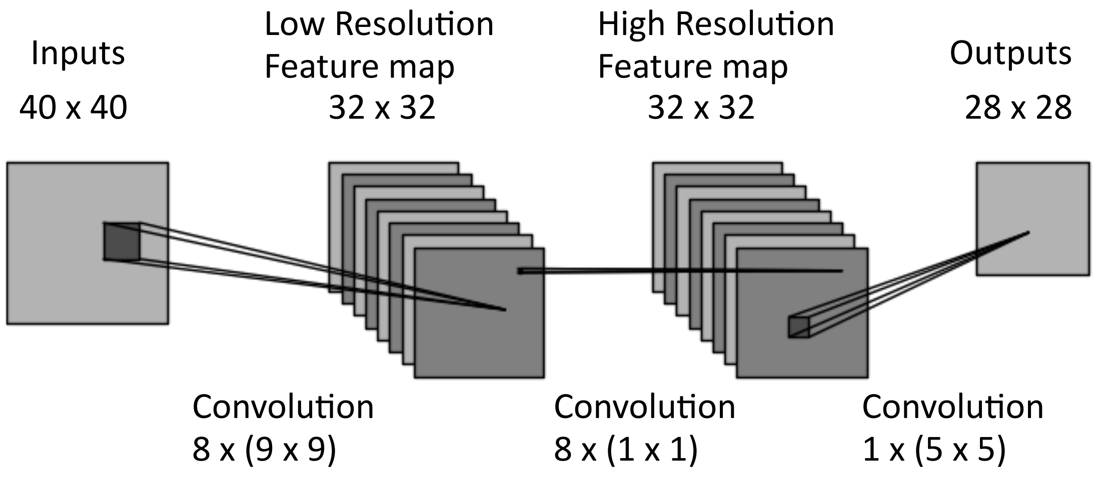

b

No.	Input	Convolutional Layer 1		Convolutional Layer 2		Convolutional Layer 3		Output
	Size	Filter Number	Filter Size	Filter Number	Filter Size	Filter Number	Filter Size	Size
1	40 x 40	8	9 x 9	8	1 x 1	1	5 x 5	28 x 28
2	40 x 40	16	9 x 9	16	1 x 1	1	5 x 5	28 x 28
3	40 x 40	8	7 x 7	8	3 x 3	1	5 x 5	28 x 28
4	40 x 40	8	9 x 9	8	3 x 3	1	3 x 3	28 x 28

c

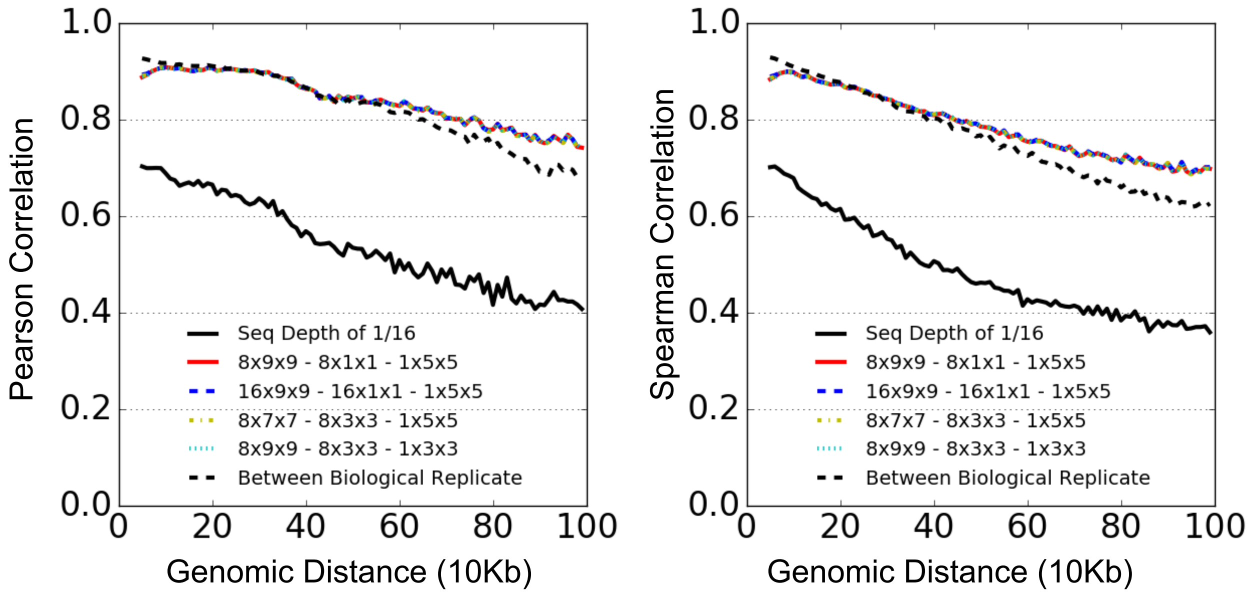

Supplementary Figure 1 | Implementation of the convolutional neural network in HiCPlus. a, The network topology of HiCPlus. HiCPlus contains three convolutional layers, and the output of the third layer is the output of overall neural network. The hyper-parameters listed here are used throughout this work unless otherwise noted. **b,** We tested a series of combinations of hyper-parameters (such as filter size and filter numbers) to study their impact. **c,** Performance comparison of different parameter selections. Similar to Fig. 2b and 2c, we computed Pearson correlations between HiCPlus enhanced matrices with original high resolution Hi-C matrix at each distance. We observe that results using four different hyper-parameter settings are indistinguishable and the performance of HiCPlus is stable.

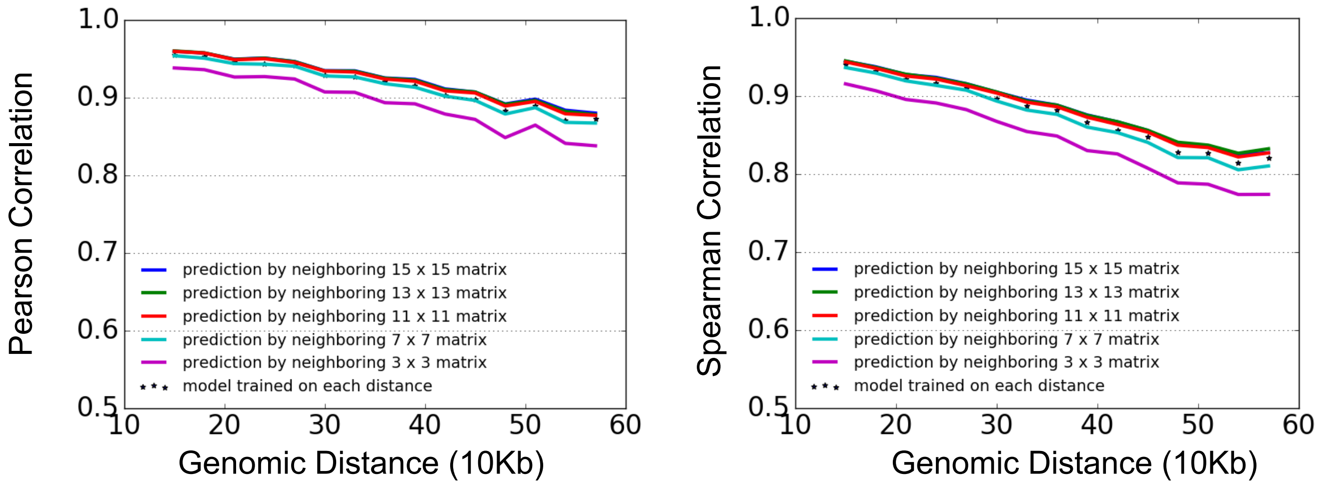

Supplementary Figure 2 | Testing the effect of using different sizes of neighbouring regions. This figure is similar to Fig. 2, but with more choices of surrounding regions (from 3x3 to 15x15). We found that there is no further improvement after 13x13 matrix and therefore we used this setting for HiCPlus throughout the manuscript. The ConvNet model is trained on chromosome 1-17 and the prediction is done in chromosome 18-22, using the 10kb resolution Hi-C data in GM12878 cells.

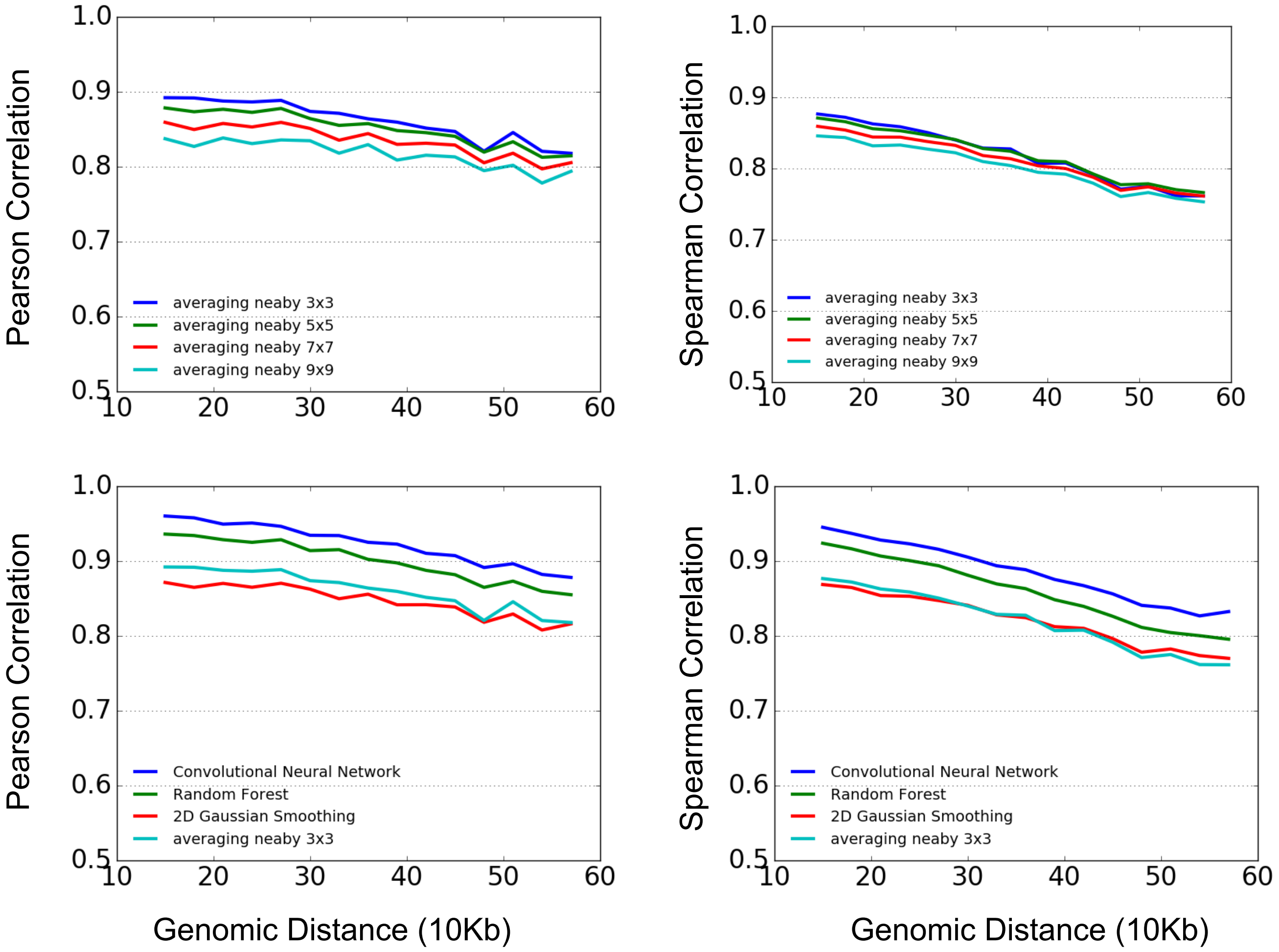

Supplementary Figure 3 | Comparing the performance of different methods to predict chromatin interactions with theirs neighbouring regions. In the upper panels, we tested the average-based method with different sizes of the neighboring cells (blocks). The results suggest that 3x3 matrix gives the best result, and therefore we used 3x3 matrix for the average-based method throughout the manuscript. In the lower panels, we compared the performance of Random Forest, 2D Gaussian Smoothing, and Convolutional Neural Network. Among them, convolutional neural network performs the best. All of the evaluations are done in chromosome 18-22, using the 10kb resolution Hi-C data in GM12878 cells. For Random Forest and Convolutional Neural Network, the model was trained in chromosome 1-17.

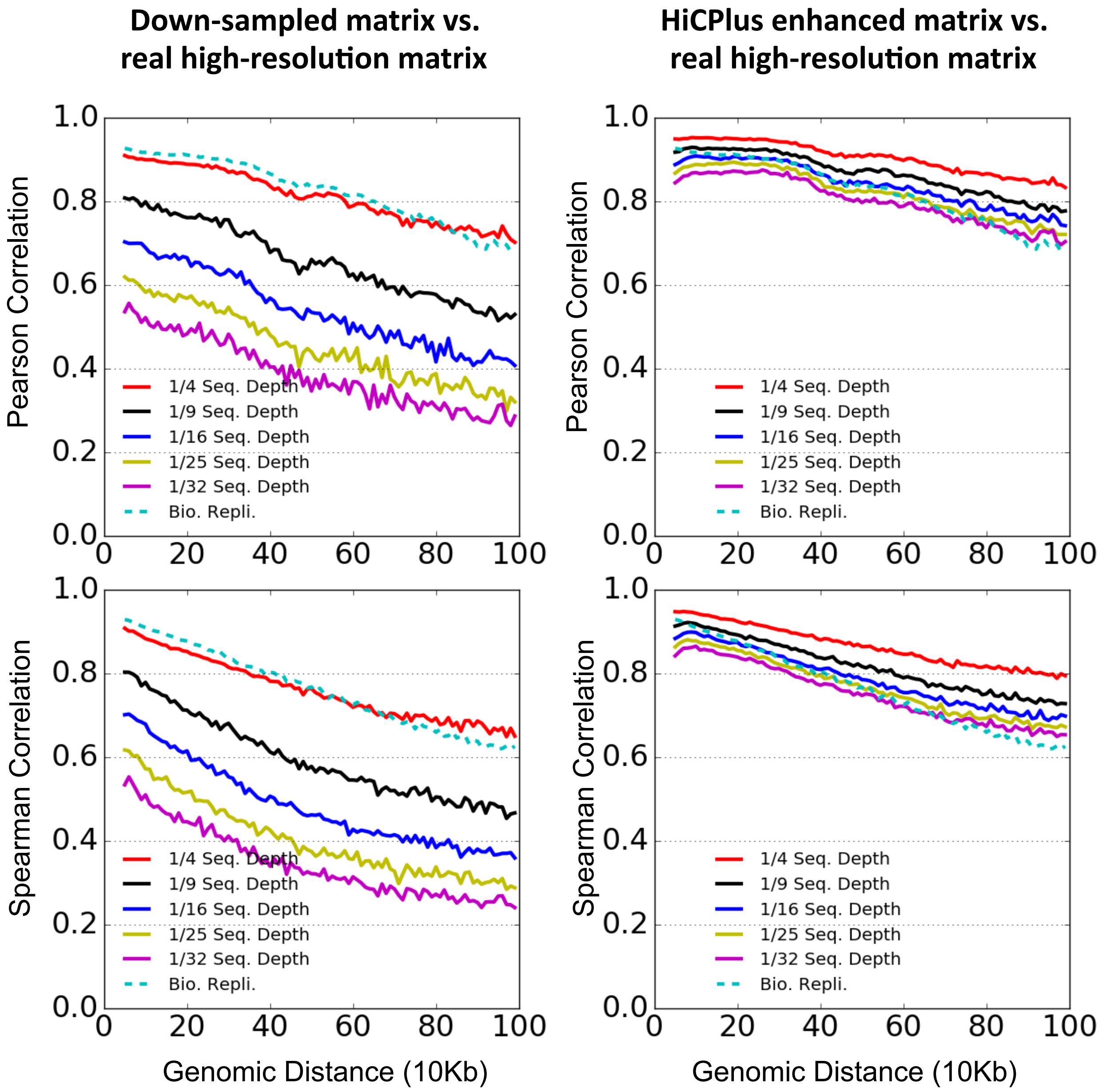

Supplementary Figure 4 | HiCPlus can generate high quality interaction matrix using a fraction of the original sequencing depth. Figures on the left column describe the correlations between down-sampled interaction matrices vs. the original high-resolution matrix. Figures on the right column describe the correlations between HiCPlus enhanced interaction matrices vs. the original high-resolution matrix. Compared with down-sampled matrix, HiCPlus significantly increased their correlation to the original deep sequenced data. We plot Pearson correlation coefficients in the top panels and Spearman correlation coefficients in the bottom panels.

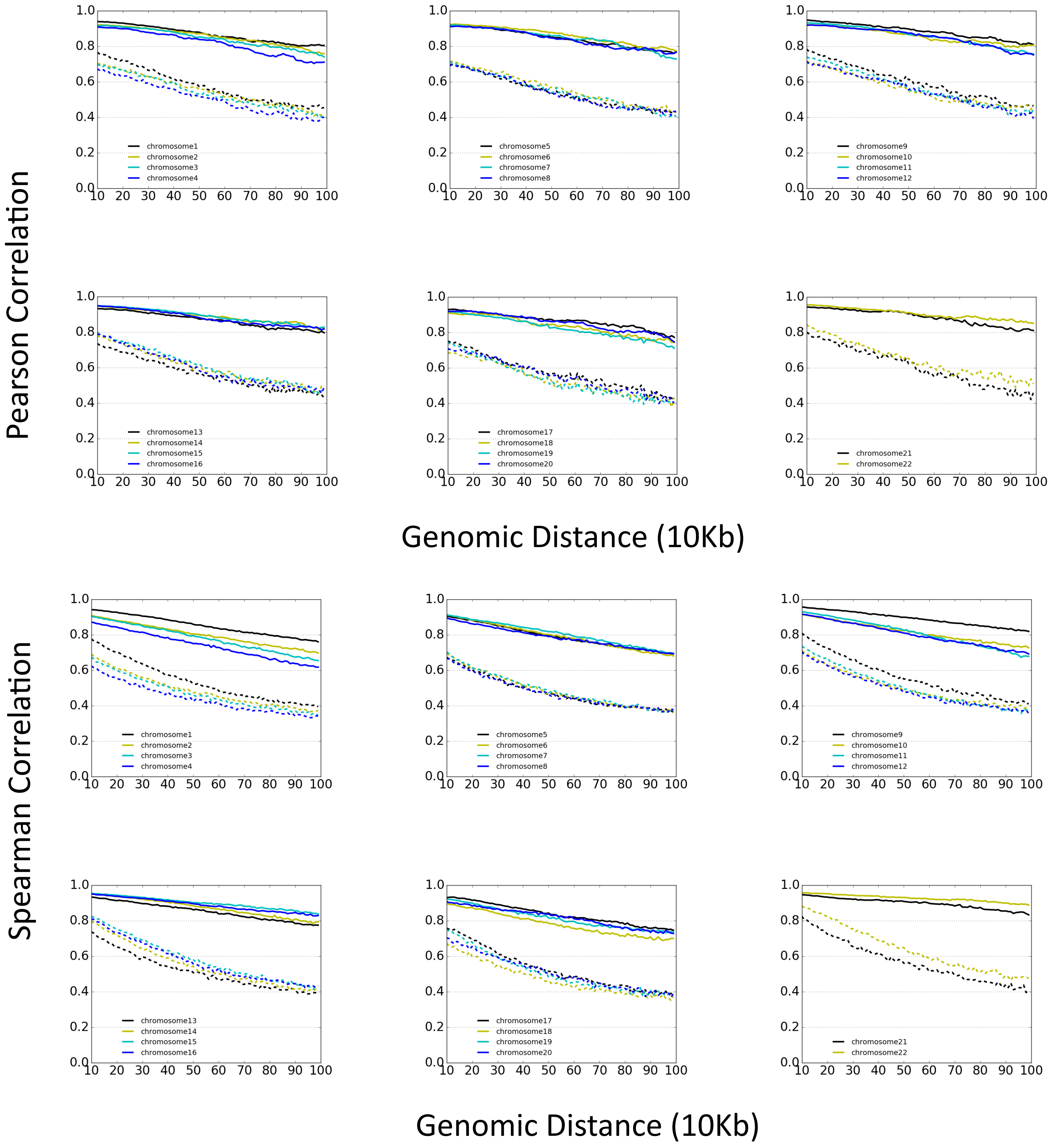

Supplementary Figure 5| The performance of HiCPlus on each chromosome. We trained the model on chr1-8 in GM cell line at 10kb resolution. We then applied the model learned in these chromosomes to systematically predict all the chromosomes, including chr1-8 themselves. In this case, the performance in chr1-8 is trained and predicted in the same datasets. More importantly, we observe the performance of prediction in chr 9 – 22 is comparable with the those in chr1-8. We also observe that the degrees of improvement of enhanced vs down-sampled matrix are also at the similar level for chr1-8 and chr9-22 and the performance of HiCPlus is consistent for each chromosome. The solid lines are the correlation coefficients between the original matrix and enhanced matrix. The dashed lines are the correlation coefficients between the original matrix and the down-sampled matrix.

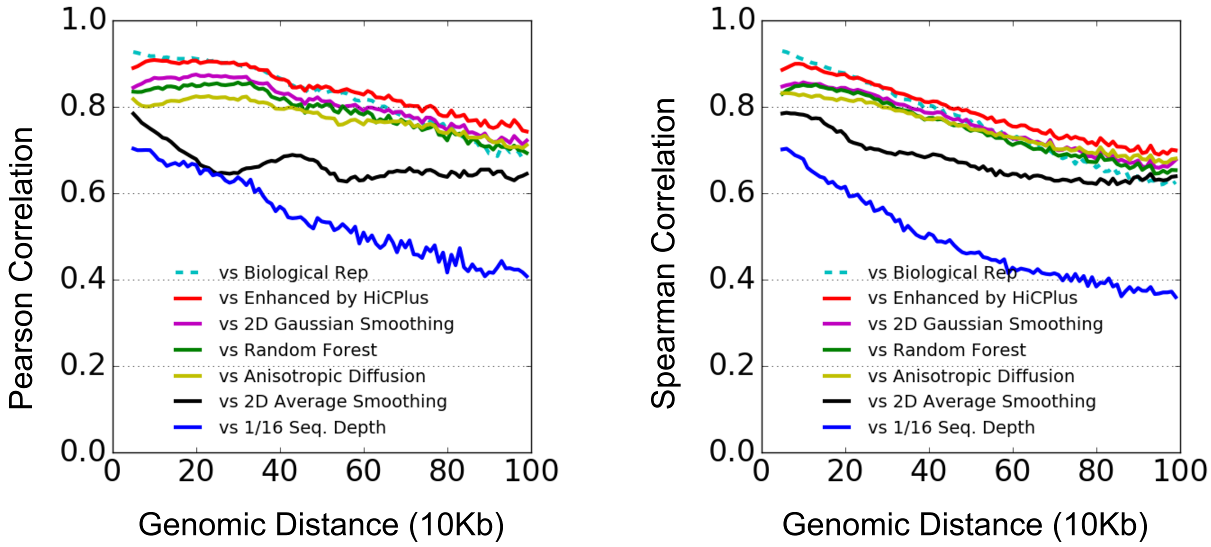

Supplementary Figure 6| Comparing the performance of HiCPlus with other methods, including Random Forest and several image denoising approaches. Here we implemented several commonly used image denoising methods, including 2D Gaussian smoothing, 2D average smoothing, and Anisotropic diffusion. Among them, 2D Gaussian smoothing has the best performance and therefore we kept it as the baseline to compare with HiCPlus. We observe that HiCPlus has the best performance, followed by 2D Gaussian Smoothing and Radom Forest. The model was trained on chromosome 1-8 and tested in chromosome 18, in the same cell type (GM12878) at 10kb resolution. We use the Python Sci-kit Learn (http://scikit-learn.org/stable/) to implement the random forest prediction. To keep the comparison fair, we used the exactly the same training data sets for both deep learning and random forest (10 kb resolution Hi-C matrix GM12878 from chr 1- 8). In the deep learning model, the X is N x 40 x 40 samples, and y is N x 28 x 28 samples. Therefore, in the non-deep learning approach, we use the same samples input and output to train the regression model. We train a total of 28 x 28 = 784 regressors to generate the enhanced Hi-C, and each regressor take 40x40 matrix (1600 attributes) as input, and output one pixel on the output matrix

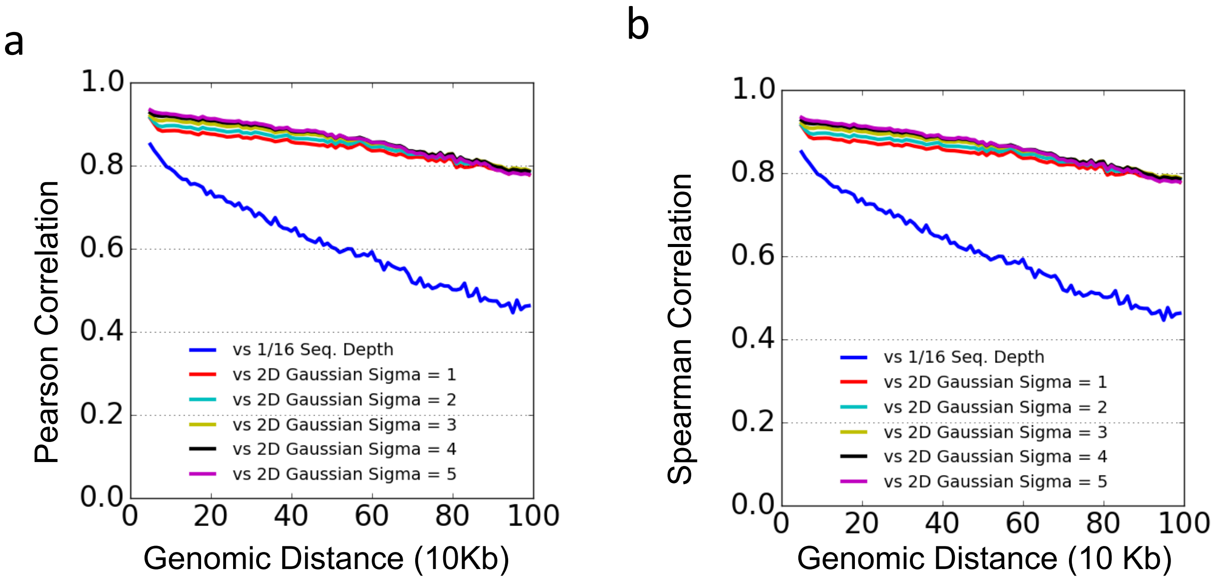

c

Sigma	1	2	3	4	5
Average Pearson Correlation	0.8391	0.8486	0.8571	0.8608	0.8606
Average Spearman Correlation	0.8685	0.8730	0.8761	0.8760	0.8736

Supplementary Figure 7 | Determining the optimal parameter for the 2D Gaussian smoothing. To determine the optimal parameter (the deviation Sigma), we tested different values for Sigma. We computed the average correlations in the distance 10-100 bins and presented the results in panel **c**. We observe that there is no further improvement after sigma=4, and therefore we use it as the optimal Gaussian kernel parameter throughout this study.

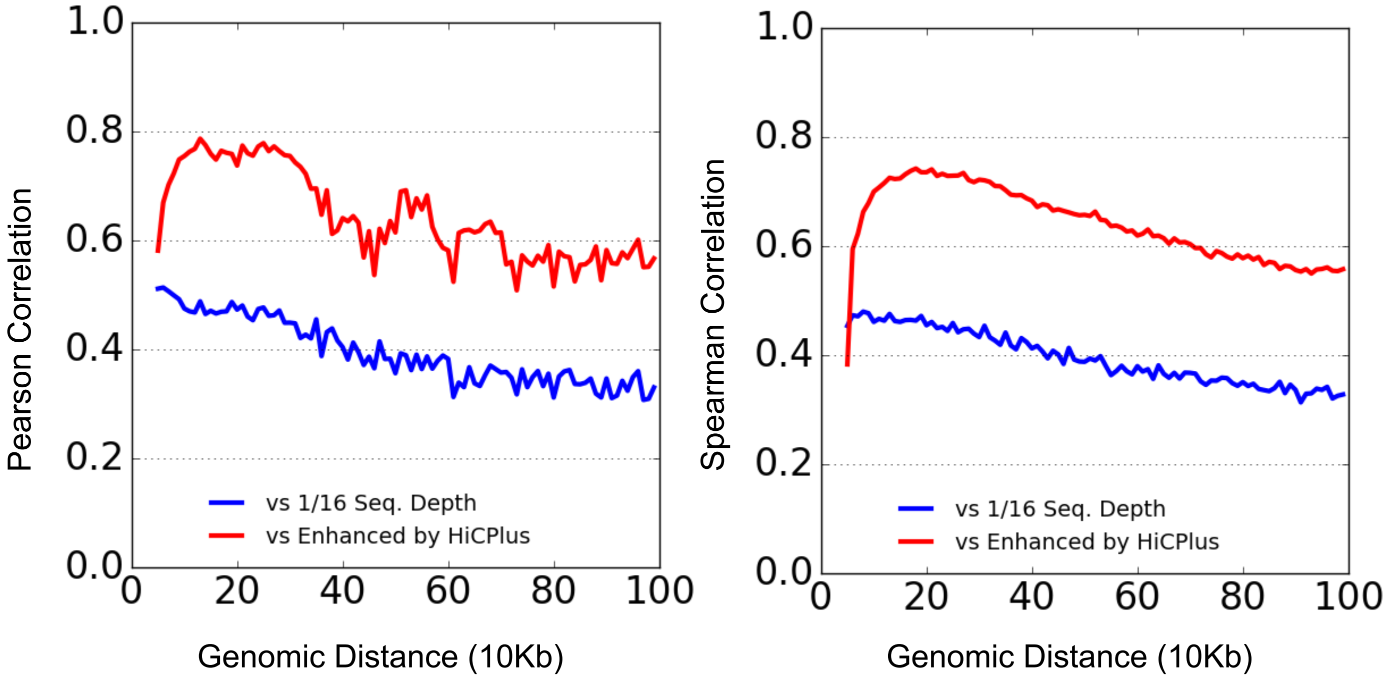

Supplementary Figure 8 | HiCPlus can also enhance normalized Hi-C interaction matrix. HiCPlus model was trained and tested with ICE normalized Hi-C data in GM12878 cells at 10kb resolution.

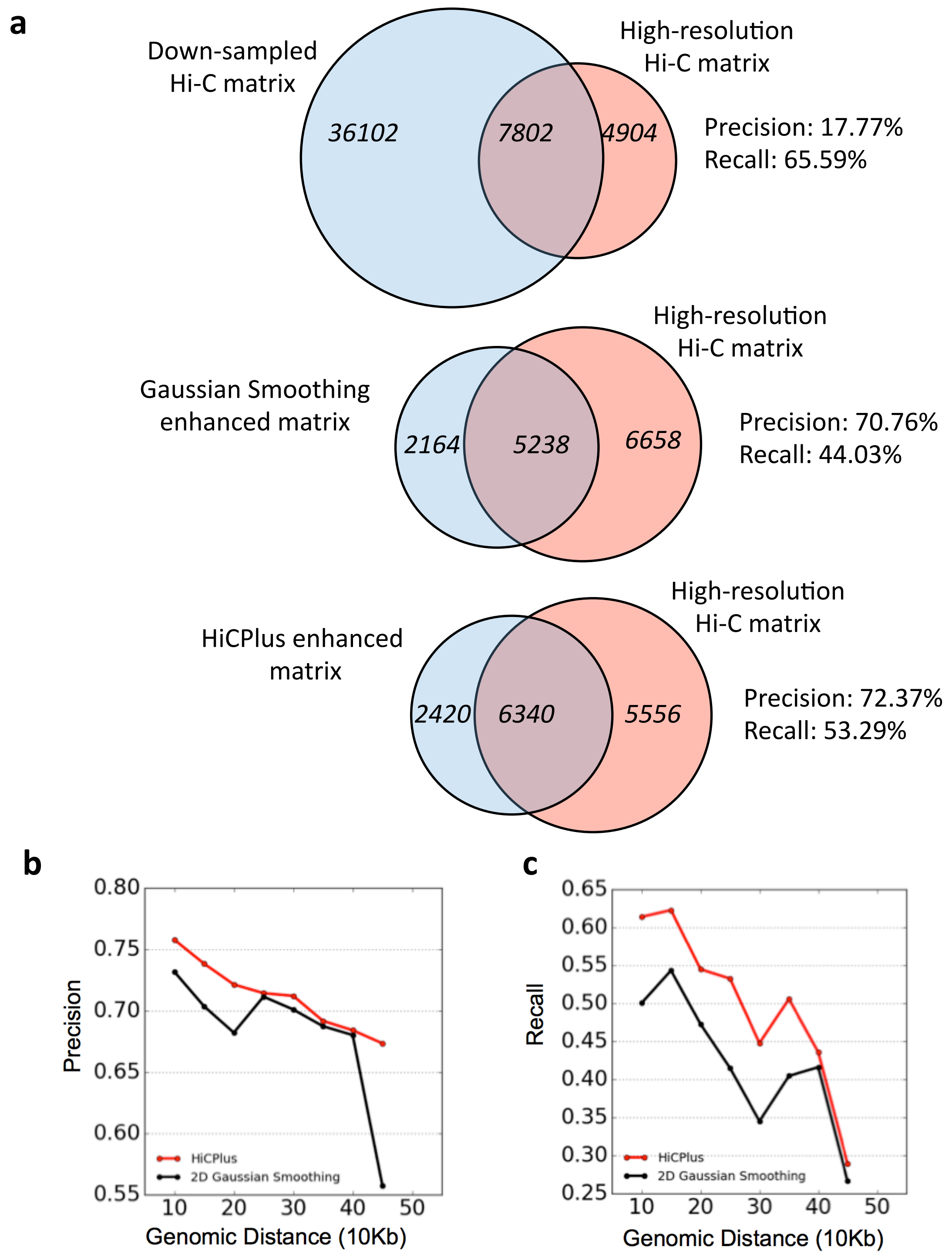

Supplementary Figure 9 | Overlap of predicted chromatin interactions from enhanced and experimental high-resolution Hi-C matrices. **a**, We used Fit-Hi-C to predict the significant interactions from the down-sampled, Gaussian smoothed and HiCPlus enhanced Hi-C matrices and compared their overlaps with the experimental high-resolution Hi-C. The red circle indicates the number of predicted chromatin interactions in the experimental high-resolution Hi-C data. We applied a stringent p-value of 10e-6 as the threshold for Fit-Hi-C. We observe that HiCPlus has a higher rate of accuracy and coverage than Gaussian smoothing matrix. **b** and **c**, Precision and recall rate of HiCPlus and Gaussian smoothing by genomic distance.

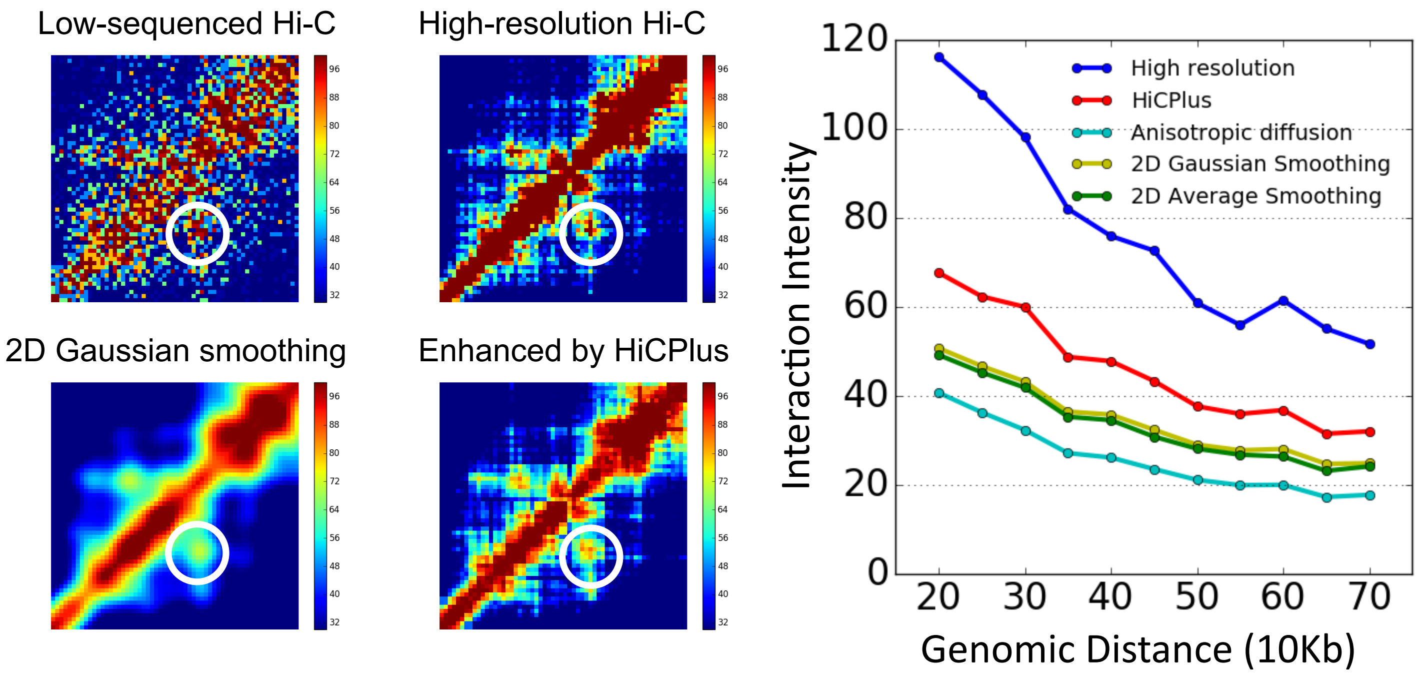

Supplementary Figure 10 | HiCPlus outperforms smoothing-based methods at chromatin interaction regions. Besides the analysis of genome-wide correlations between the enhanced and original matrices, we also investigated their performance on the predicted chromatin loops. **a,** We noticed that both Gaussian smoothing and HiCPlus performed well for noise reduction from the low-resolution Hi-C matrix (Chr18:33.5M-34.1M). However, we observe that in the Gaussian smoothed Hi-C matrix, the chromatin interaction intensity at chromatin loop region (marked by white circle) is much weaker than the value in the high-resolution matrix, and also harder to distinguish from its neighbors. The matrix enhanced by HiCPlus, on the other hand, is more similar to the high-resolution Hi-C matrix and the chromatin loop is more visible. **b,** We compared the frequencies at chromatin interaction peaks identified in the original high-resolution Hi-C matrix. We found that interaction intensities predicted by HiCPlus are closer to real Hi-C data than the matrices enhanced by other methods. Y-axis indicts the average interaction intensities for chromatin loops identified at each genomic distance.

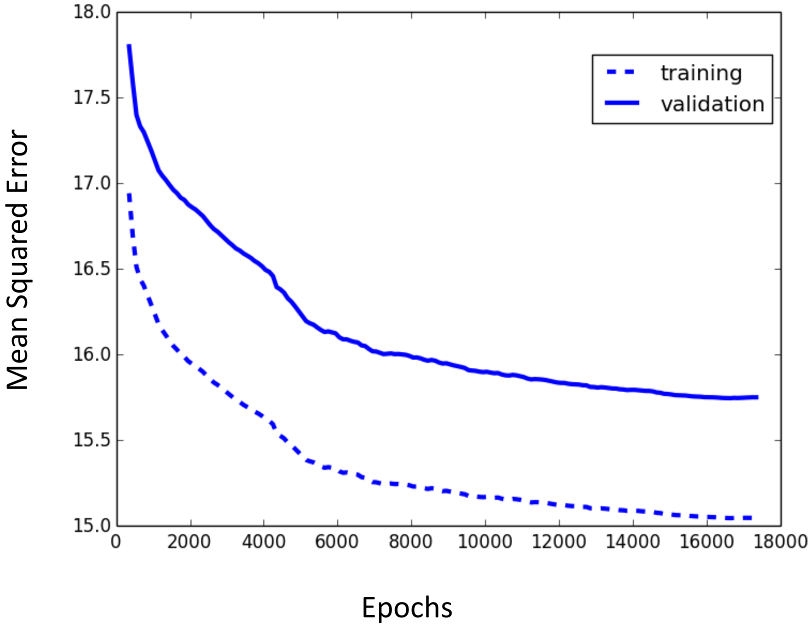

Supplementary Figure 11 | Estimation of potential over-fitting in HiCPlus model. To study the possible over-fitting issue in our model, we calculated Mean Squared Error (MSE), during the training process on the training sets (chromosome 1-8) and validation sets (chromosome 19-22) in GM12878 cell line. We observe that the loss in training and training keep the same trend in the entire training process.

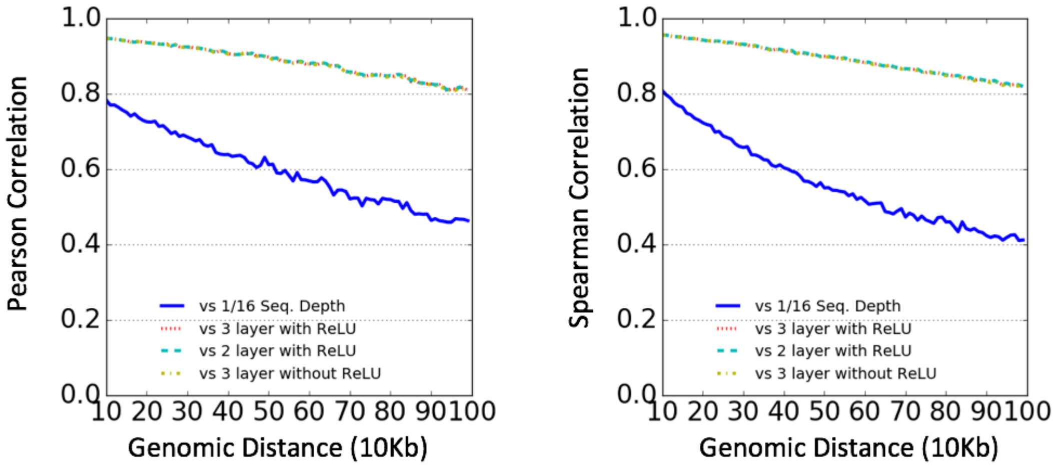

Supplementary Figure 12 | Comparing the performance of convolutional neural network with two layers, three layers, and three layers without ReLU activation. We found the results of three different settings are highly similar.

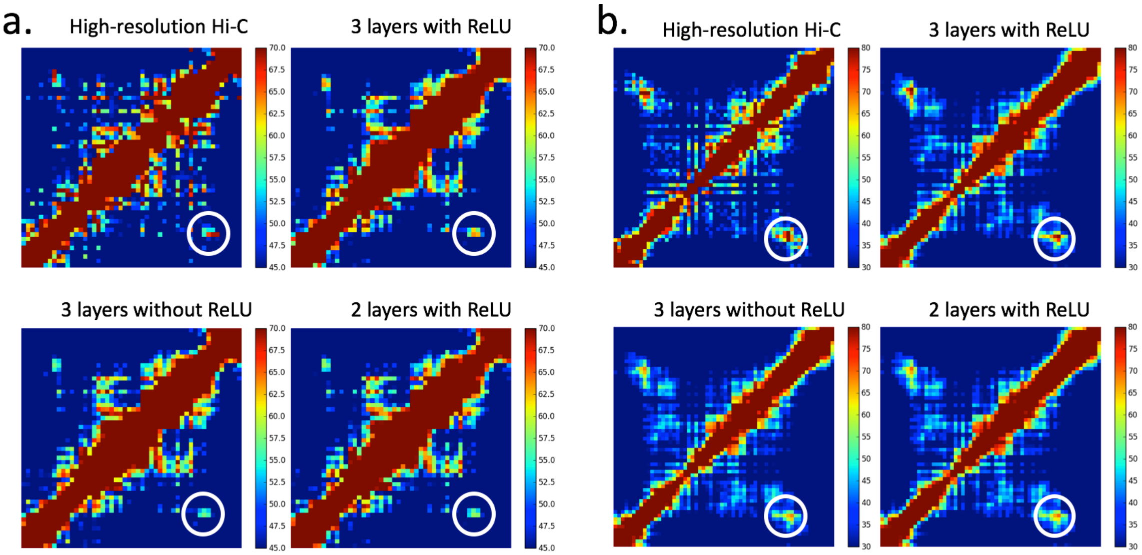

Supplementary Figure 13 | Comparing the performance of convolutional neural network with different hyper-parameters at chromatin loop regions. We compare the performance of convolutional neural network with different hyper-parameters at the loop region (a: Chr18:33.5M-34.1M. b: Chr9:5.0M-5.5M). We noticed that compared to the network with 3-layer and ReLU activation, the other two models show reduced chromatin intensities at chromatin loop regions.

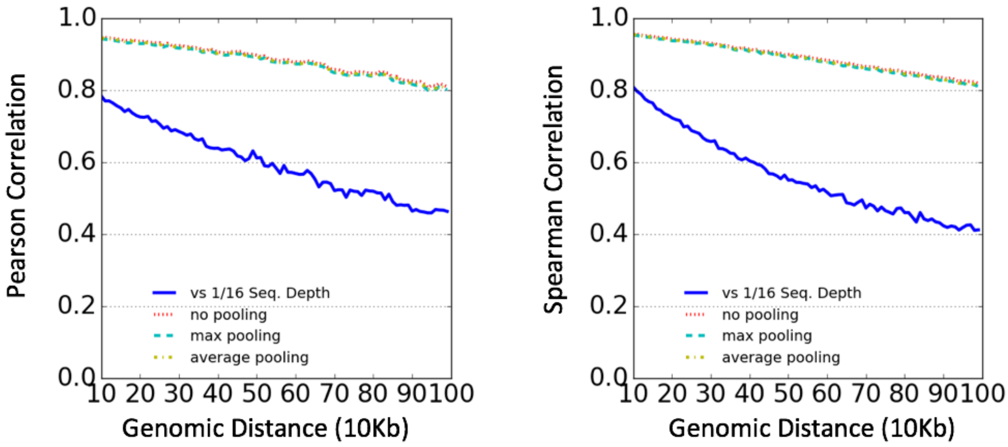

Supplementary Figure 14 | We compared the performance of convolutional neural network without pooling (current HiCPlus implementation), max pooling and average pooling. We did not observe any performance gain using a pooling layer. .

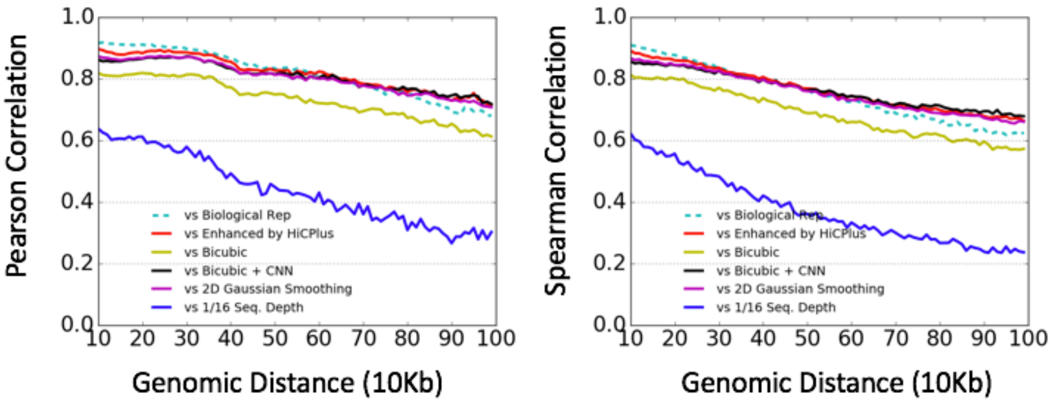

Supplementary Figure 15 | Genome-wide comparison of the performance of low-resolution and low-resolution interpolated matrix. We found simple bicubic interpolation (yellow line) and the bicubic interpolation followed by ConvNet (black line) both have good performance, but not as good as HiCPlus.

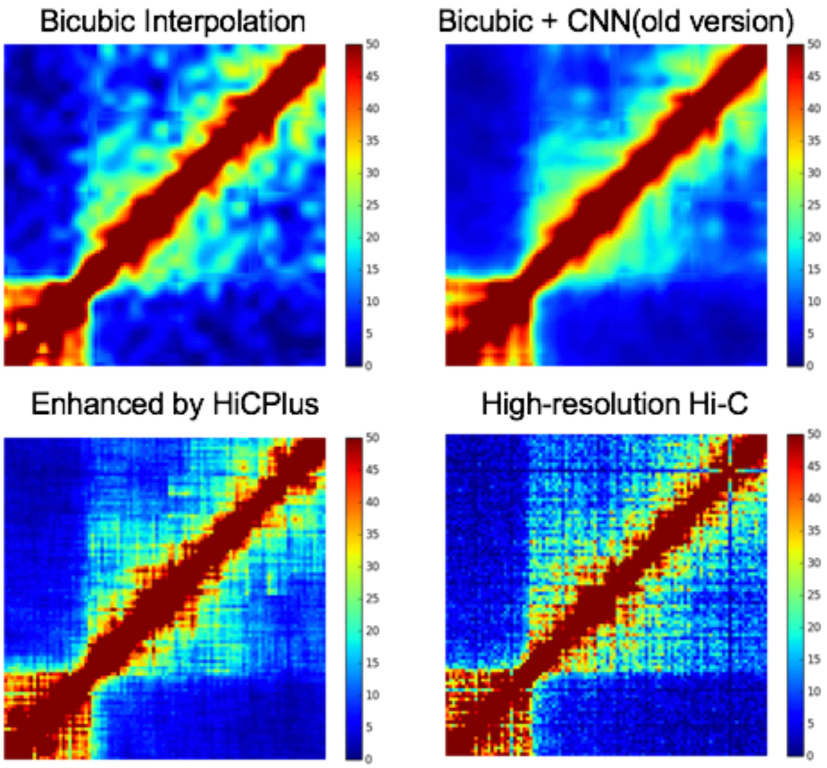

Supplementary Figure 16 | An example to compare the performance of using direct interpolated matrix, low-resolution interpolated matrix followed by convolution neural network, and HiCPlus in region Chr18:4.8-4.9M. We found the result of the HiCPlus is the most similar to the real high-resolution Hi-C matrix.

Supplementary Table 1: List of tissue/cell types where we applied HiCPlus to enhance the Hi-C data resolution.

Tissue/Cell type	Dataset	Model selection/ Enhancement ratio
Psoas	PO3	16
Spleen	SX3	16
Pancreas	PA3	16
Lung	LG2	16
Lung	LG1	16
Psoas	PO1	16
Ovary	OV2	16
Small Bowel	SB2	16
Pancreas	PA2	16
Adrenal	AD2	16
Spleen	SX1	16
Bladder	BL1	16
Hippocampus	Hippo	16
Neural Progenitor Cell	Npc_rep1	9
Right Ventricle	STL003	9
Neural Progenitor Cell	Npc_rep2	9
Aorta	STL002	4
Trophoblast-like Cell	Tro_rep1	4
Trophoblast-like Cell	Tro_rep2	4
Liver	STL011	4

Supplementary Table 2: Comparison of interactions identified in the experimental high-resolution and enhanced matrices.

<table>
<tr><th></th><th>Predicted Interactions by FitHiC</th><th>Overlap with experimental Hi-C</th><th>Predicted Interactions in experimental Hi-C</th><th>Accuracy</th><th>Coverage</th></tr>
<tr><td>Low-sequencing depth Hi-C</td><td>43,904</td><td>7,802</td><td rowspan="3">11,896</td><td>17.77%</td><td>65.59%</td></tr>
<tr><td>Gaussian Smoothing enhanced</td><td>7,402</td><td>5,238</td><td>70.76%</td><td>44.0%</td></tr>
<tr><td>HiCPlus enhanced</td><td>8,760</td><td>6,340</td><td>72.37%</td><td>53.29%</td></tr>
</table>
